# Decreased surface receptors, function, and suboptimal osteoclasts-induced cell expansion in natural killer (NK) cells of elderly subjects

**DOI:** 10.18632/aging.206226

**Published:** 2025-03-26

**Authors:** Kawaljit Kaur, Anahid Jewett

**Affiliations:** 1 Division of Oral Biology and Medicine, The Jane and Jerry Weintraub Center for Reconstructive Biotechnology, University of California School of Dentistry, Los Angeles, CA 90095, USA; 2 The Jonsson Comprehensive Cancer Center, UCLA School of Dentistry and Medicine, Los Angeles, CA 90095, USA

**Keywords:** NK cells, osteoclasts, supercharged NK cells, expansion, IFN-γ

## Abstract

Natural killer (NK) cells are known for their cytotoxic and cytokine secretion capabilities. The balance of activating and inhibitory receptors on their surface regulates NK cell function and survival. However, it is not fully understood how aging may modulate the levels of NK cell surface receptors ultimately affecting their interaction with other immune cells, especially with those known to activate and expand NK cells. Here, we report decreased levels of NK cells’ surface receptors, cytotoxic function, and cytokine secretion in aged donors (75-85 years) as compared to younger donors (21-25 years). We used our previously established methodology to expand and supercharge NK cells from young and older individuals using osteoclasts (OCs) and probiotic bacteria. Significantly lower levels of NK cell expansion and functional activation were seen in NK cells from 75-85-year-old donors when compared to younger donors’ NK cells. Surface receptors of OCs were also found to be decreased in 75-85-year-old donors compared to younger donors. In addition, OCs from 75-85-year-old donors induced lower levels of cell expansion and functional activation of NK cells when compared to OCs from younger donors. These findings illustrate defects in both peripheral blood-derived primary NK cells and OCs in older individuals; however, suppression appears to be more in NK cells when compared to OCs.

## INTRODUCTION

NK cells are cytotoxic lymphocytes, that can recognize and lyse tumor cells and virally infected cells without prior sensitization [1]. Human NK cells are identified by their CD16 and CD56 surface receptors, and are activated by several different cytokines [2, 3]. We have previously demonstrated that NK cells play a crucial role in limiting the survival and expansion of cancer stem-like cells (CSCs) via direct killing or the induction of differentiation, through their secreted IFN-γ and TNF-α [4–6]. The functional state of NK cells is associated with infection and disease control and prognosis. NK cell dysfunction was found to be associated with the onset of autoimmune diseases [7]. Modulation of NK cell number or function impacts the overall immune system [8].

Aging was found to profoundly impact innate immunity [9]. Age-associated modulation in NK cell number, phenotype, and function was found to be directly attributed to several diseases and infections [10–13]. Decreased NK cell number and function were found to be associated with increased susceptibility to infection, cancer, autoimmune diseases, coronary heart diseases, liver fibrosis, and neurodegenerative diseases in elderly people [8, 14–17]. Levels of cytokines especially IFN-γ were found to be significantly lower in NK cells from older individuals [18]. NK cell proliferation was found to decline in the elderly population [10]. Chuang Guo et al., have demonstrated that the CD52+ NK cells subset predominately accumulated in the elderly population and exhibited proinflammatory characteristics contributing to the spread of infections [19].

In this study, we compared the phenotype, function, and expansion of NK cells from the young and older individuals. We have previously reported the methodology to activate and expand NK cells using a combination of osteoclasts (OCs) and probiotic bacteria [20–41]. In the current study, we compared the phenotype and function of OC-expanded NK cells of young and older donors using allogeneic and autologous OCs. We found lower expansion and functional activation in NK cells from older individuals using allogeneic young OCs or autologous OCs.

## RESULTS

### Suppression of surface receptors, cytotoxicity, and secretion of IFN-γ in NK cells of older human donors

**Figure 1:**
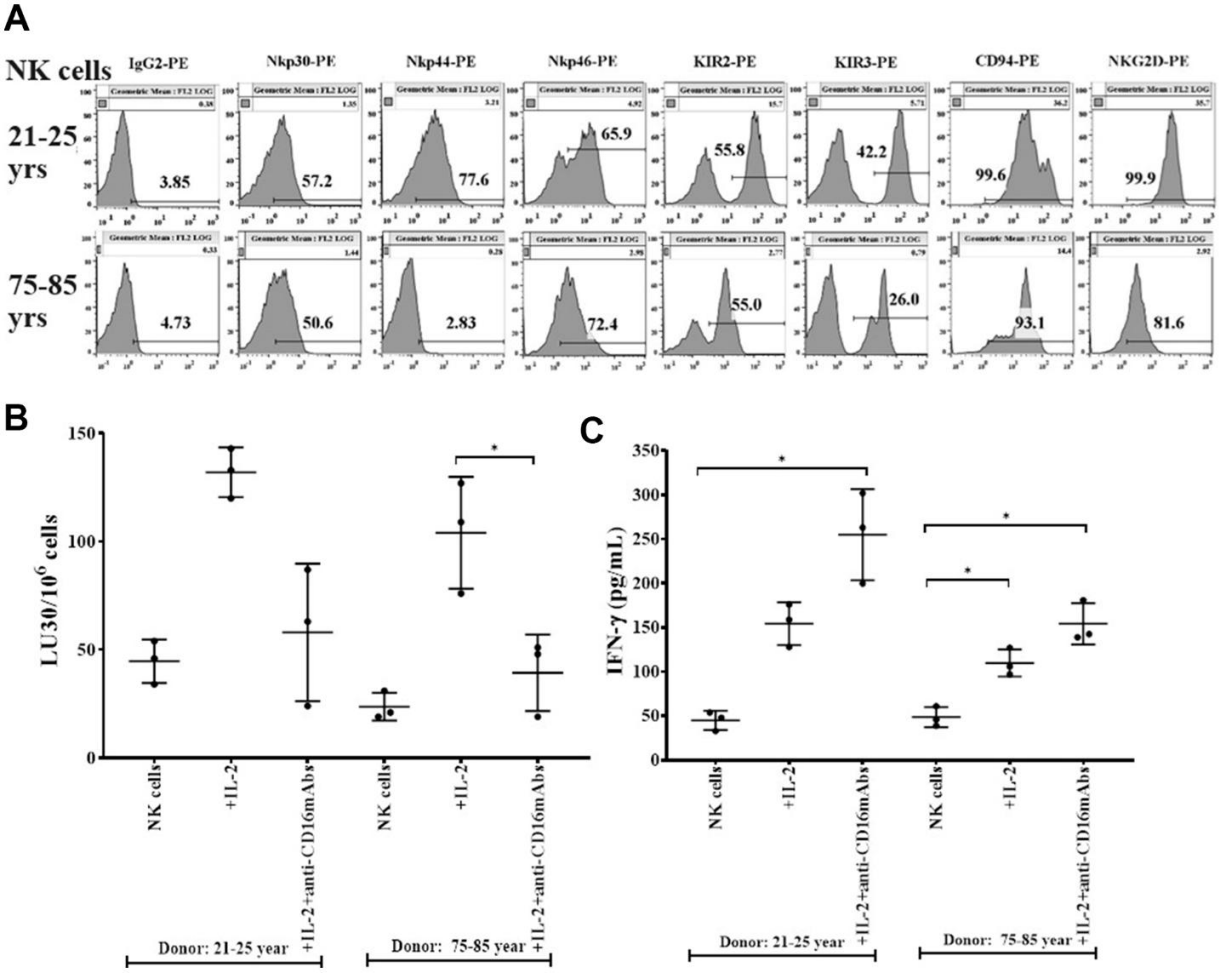
**Decreased levels of surface receptors, cytotoxicity, and IFN-γ secretions in NK cells from older donors.** PBMCs-derived NK cells from 21-25 years and 75-85 years donors were used to determine the surface expression levels of Nkp30, Nkp44, Nkp46, KIR2, KIR3, CD94, and NKG2D using flow cytometric analysis (one of three representative experiments is shown in the figure) (**A**). NK cells were left untreated or were treated with IL-2 (1000 U/ml) or with a combination of IL-2 (1000 U/ml) and anti-CD16 mAbs (3 μg/ml) for 18 hours before they were used as effectors in standard 4-hour ^51^Cr release assay against OSCSCs (n=3, **B**). The lytic units 30/10^6^ cells were determined using the inverse number of NK cells required to lyse 30% of OSCSCs X 100. NK cells left untreated ot treated with IL-2 (1000 U/ml) or with a combination of IL-2 (1000 U/ml) and anti-CD16 mAbs (3 μg/ml) for 18 hours before the supernatants were harvested to determine IFN-γ secretion using single ELISA (n=3, **C**). *(*p*-value 0.01-0.05).

Lower surface expression of Nkp30, Nkp44. KIR2, KIR3, CD94, and NKG2D, and slightly higher surface expression of Nkp46 were observed in NK cells of 75-85 years donors when compared to donors of 21-25 years of age (Figure 1A). Nkp30, Nkp44, Nkp46 and NKG2D are activating receptors and KIR2, KIR3, CD94/NKG2A are inhibitory receptors. Balance of these activating and inhibitory receptors plays crucial role for NK cell for NK cell function. Indeed, imbalance or modulation of these receptors may result in the defective functional activity and cell expansion of NK cells. When function of NK cells was assessed, NK cells from 75-85 years donors mediated lower cytotoxicity (Figure 1B and Supplementary Table 1A) and secreted lower amounts of IFN-γ (Figure 1C and Supplementary Table 1B) compared to NK cells of 21-25 years donors.

### Suboptimal cell expansion was observed in OC-expanded NK cells of older donors compared to 21-25 years donors

**Figure 2:**
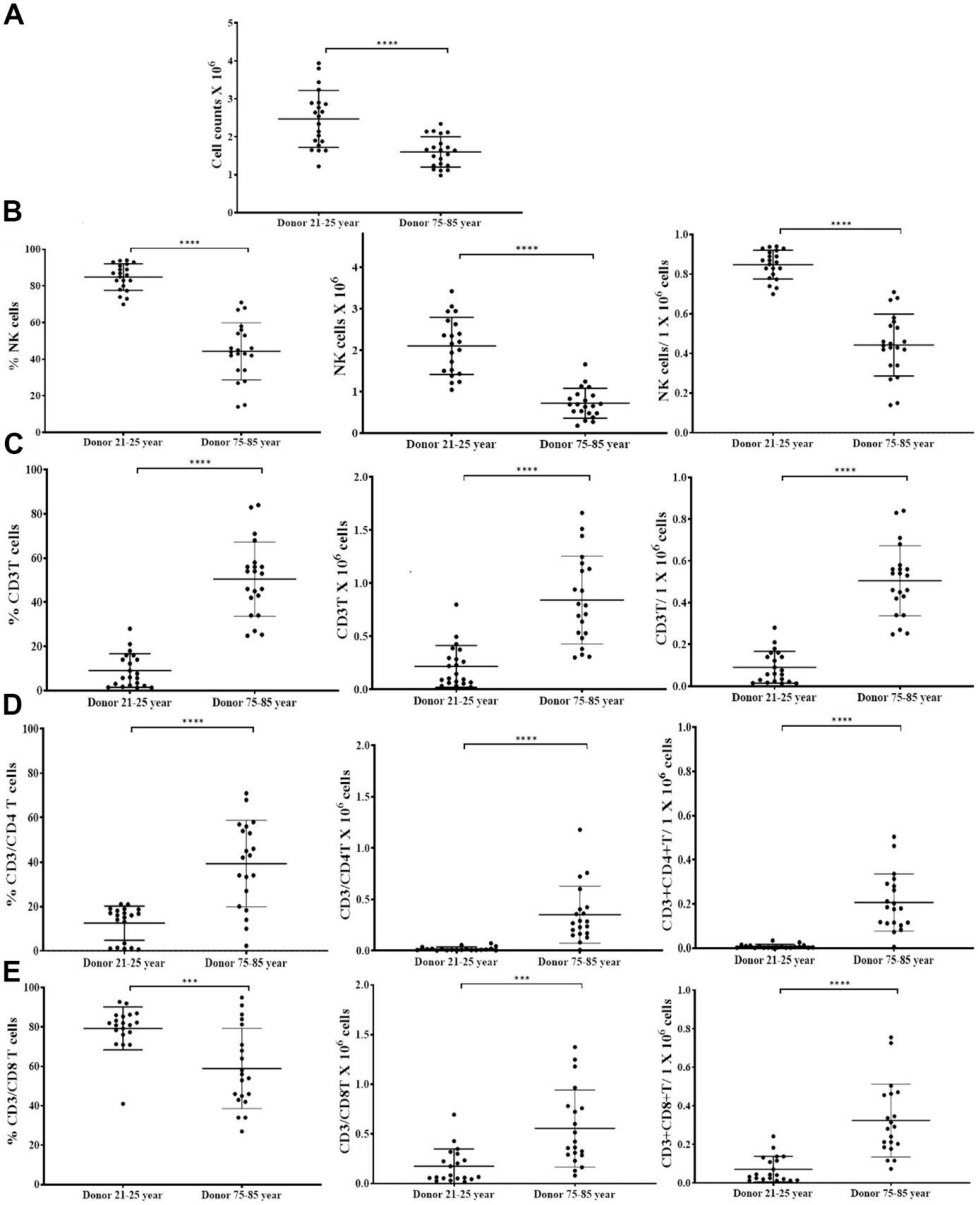
**OCs induced lower levels of cell expansion in old-age donor NK cells.** Osteoclasts (OCs) were generated as described in the Materials and Methods section. NK cells (0.5x10^6^ cells/ 2ml) were treated with a combination of IL-2 (1000 U/ml) and anti-CD16mAbs (3 g/ml) for 18 hours before they were co-cultured with OCs and probiotic bacteria sAJ2 (1:2:4: OCs:NK:sAJ2). NK cells were counted on days 6, 9, 12, 15, 18, 21, and 25, the day 0 counts were 0.5x10^6^ cells/2 ml, and 0.5x10^6^ cells/2 ml were cultured every 3 days (n=27, **A**). CD16+CD56+ NK cells, number of NK cells, and NK cells per one million of total expanding cells (**B**), CD3+ T cells, number of T cells, and T cells per one million of total expanding cells (**C**), CD3+CD4+ T cells, number of CD4+ T cells, and CD4+ T cells per one million of total expanding cells (**D**), CD3+CD8+ T cells, number of T cells, and CD8+ T cells per one million of total expanding cells (**E**), were determined on days 6, 9, 12, 15, 18, 21, and 25 (n=27). ****(p-value <0.0001), ***(*p*-value 0.0001-0.001).

To determine the extent of cell expansion and functional activation, we co-cultured NK cells with allogeneic OCs from young donors in the presence of probiotic bacteria. NK cells were treated with IL-2 and anti-CD16 mAbs for 18 hours followed by co-culture with OCs in the presence of probiotic bacteria sAJ2 as described in our previous publications [4, 36, 42]. NK cells from 75-85 years donors showed significantly decreased levels of cell expansion (Figure 2A and Supplementary Figure 1A). We have also observed previously that the small number of contaminants of T cells in purified population of NK cells from cancer patients can also expand during supercharging of the NK cells [28, 36]. Here, we determined the subpopulations of CD16+CD56+ NK cells, CD3+CD16+CD56+ NKT cells, CD3+ T cells, CD3+CD4+ T cells, and CD3+CD8+ T cells within the expanding NK cell cultures from the elderly individuals. (Figure 2B–2E and Supplementary Figure 1B–1L). No significant differences were seen in percentages and counts of NKT cells when compared between 75-85-year-old donors and 21-25-year-old donors expanding NK cell cultures (Supplementary Figure 1B–1D). Significantly lower percentages and decreased counts of NK cells (Figure 2B and Supplementary Figure 1E, 1F), and higher percentages and counts of T cells, CD3+CD4+ T cells, and CD3+CD8+ T cells (Figure 2C–2E and Supplementary Figure 1G–1L) were observed in NK cell expanding cultures of 75-85 years donors when compared to 21-25 years donors.

### Suppressed cytotoxicity and secretion of IFN-γ was observed in OC-expanded NK cells of older age donors compared to 21-25 years donor

**Figure 3:**
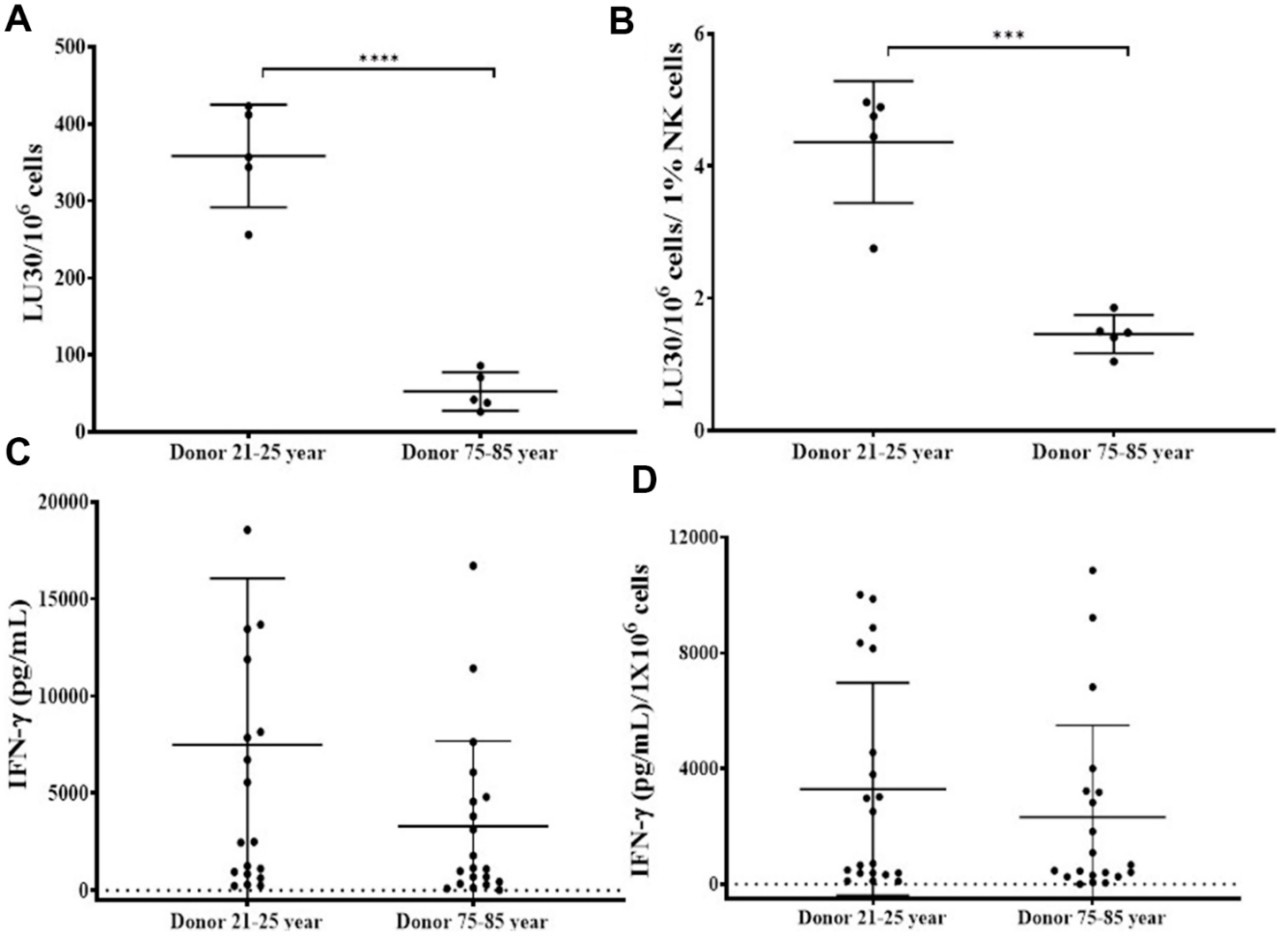
**OCs induced lower levels of activation in old-age donor NK cells.** Osteoclasts (OCs) were generated as described in the Materials and Methods section. NK cells and OCs co-culture was performed as described in Figure 2. NK cell-mediated cytotoxicity against OSCSCs was determined on days 9 and 15 using a standard 4-hour ^51^Cr release assay. The lytic units 30/10^6^ cells were determined using the inverse number of NK cells required to lyse 30% of OSCSCs x 100 (**A**). Lytic units per 1 % NK cells were determined based on the percentages of CD16+/CD56+ NK cells in the cultures obtained by flow cytometric analysis (**B**). The supernatants were harvested from the cultures on days 6, 9, 12, 15, 18, 21, and 25 to determine IFN-γ secretion using single ELISA (**C**), and the levels were adjusted based on per million of NK cells (**D**). ****(p-value <0.0001), ***(*p*-value 0.0001-0.001).

NK cells of 75-85 years donors and 21-25 years donors were cultured with allogeneic OCs (23-25 years donors) as described in Figure 2. OC-expanded NK cells from 75-85 years donors showed significantly decreased levels of NK cell-mediated cytotoxicity (Figure 3A, 3B and Supplementary Table 2), and secretion of IFN-γ (Figure 3C, 3D and Supplementary Figure 2) compared to those of 21-25 years donors.

### Suppressed surface receptor ligands on osteoclasts of 75-85 years donors

We analyzed OCs surface ligands that interact with NK cell receptors during NK and OCs interaction. Reduced expression of MHC class I, CD54, KIR2, KIR3, KLRG1 and MIC A/B were observed on osteoclasts from older age donors. Reduced expression levels of MHC-class I, CD54, KIR2, KIR3, KLRG1, and MICA/B on osteoclasts from old age donors (Supplementary Figure 3).

### Osteoclasts from older individuals induced lower levels of cell expansion in NK cells

**Figure 4:**
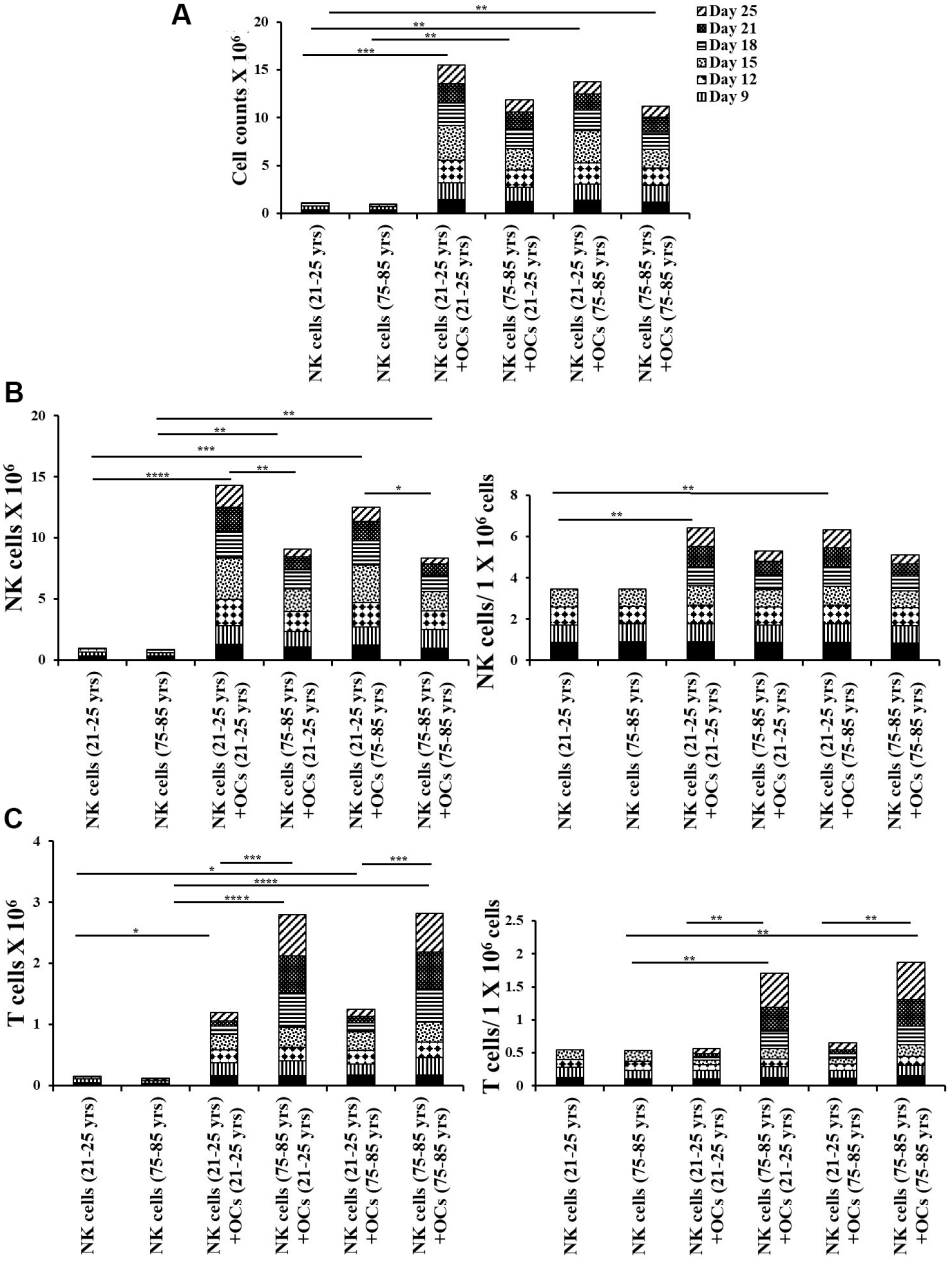
**Old-age donor-derived OCs induced lower levels of cell expansion in NK cells.** Osteoclasts (OCs) were generated as described in the Materials and Methods section. NK cells (0.5x10^6^ cells/ 2ml) were treated with a combination of IL-2 (1000 U/ml) and anti-CD16mAb (3μg/ml) for 18 hours before they were co-cultured with criss-cross OCs in the presence of probiotic bacteria sAJ2 (1:2:4: OCs:NK:sAJ2). NK cells were counted on days 6, 9, 12, 15, 18, 21, and 25, the day 0 counts were 0.5x10^6^ cells/2 ml, and 0.5x10^6^ cells/2 ml were cultured every 3 days (n=7, **A**). Number of NK cells, and NK cells per one million of total expanding cells (**B**) and the number of T cells, and T cells per one million of total expanding cells (**C**) were determined on days 6, 9, 12, 15, 18, 21, and 25 (n=27). ****(p-value <0.0001), ***(*p*-value 0.0001-0.001).

**Figure 5:**
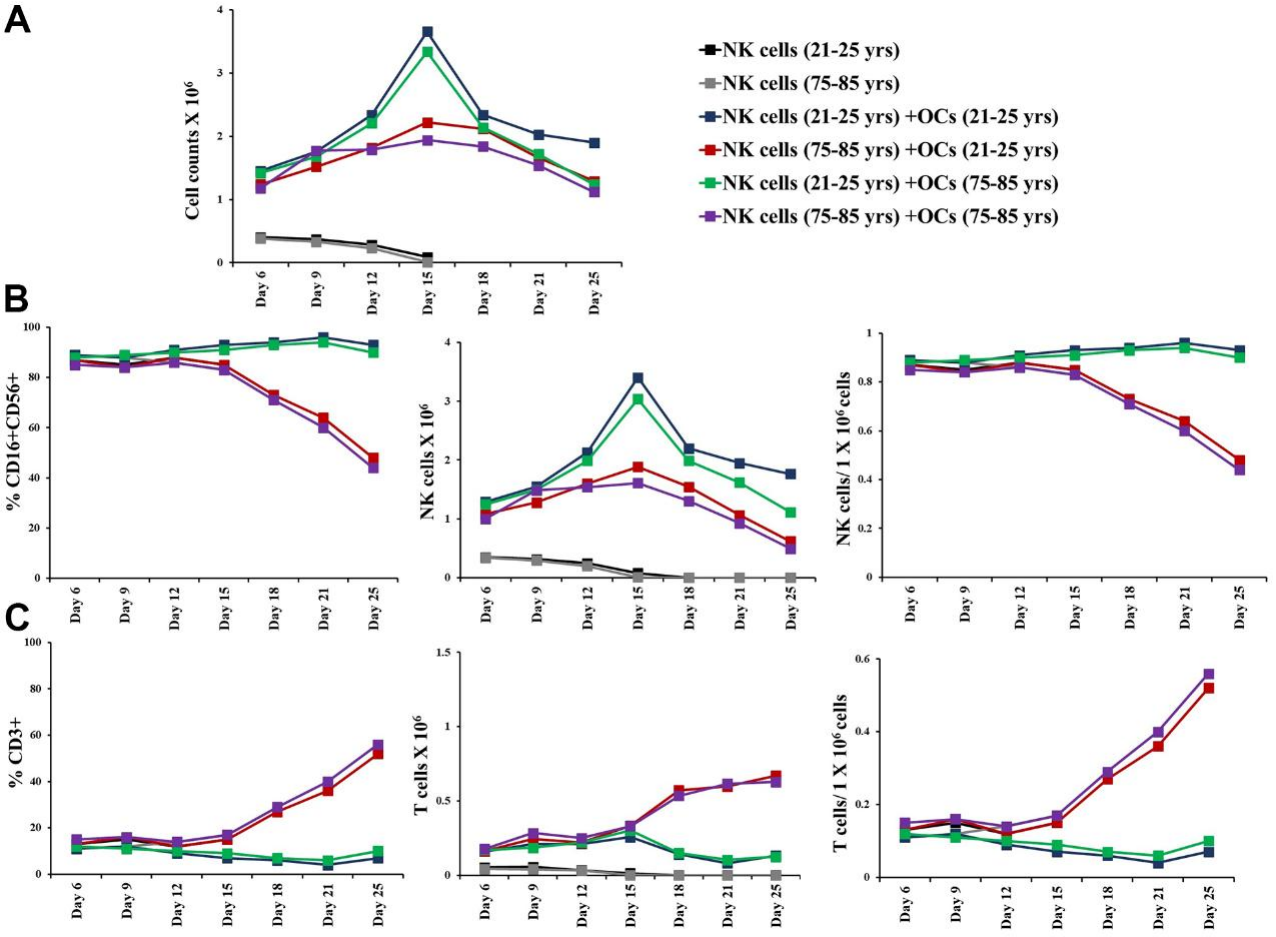
**Older individual-derived OCs induced lower levels of cell expansion in NK cells.** Osteoclasts (OCs) were generated as described in the Materials and Methods section. NK cells (0.5x10^6^ cells/ 2ml) were treated with a combination of IL-2 (1000 U/ml) and anti-CD16mAb (3μg/ml) for 18 hours before they were co-cultured with criss-cross OCs in the presence of probiotic bacteria sAJ2 (1:2:4: OCs:NK:sAJ2). NK cells were counted on days 6, 9, 12, 15, 18, 21, and 25, the day 0 counts were 0.5x10^6^ cells/2 ml, and 0.5x10^6^ cells/2 ml were cultured every 3 days (n=27, **A**). CD16+CD56+ NK cells, number of NK cells, and NK cells per one million of total expanding cells (**B**) and CD3+ T cells, number of T cells, and T cells per one million of total expanding cells (**C**) were determined on days 6, 9, 12, 15, 18, 21, and 25 (n=27). ****(p-value <0.0001), ***(*p*-value 0.0001-0.001).

The co-cultures of IL-2+anti-CD16 mAbs treated NK cells and OCs in the presence of probiotic bacteria were performed. NK cells and OCs were co-cultured in a criss-cross manner in these experiments (Figure 4). OCs from old age donor induced lower levels of cell expansion in NK cells both from young and old-age donors. (Figures 4A, 5A). When we determined NK and T cells populations in expanding NK cells, the lower levels of NK cell population (Figures 4B, 5B) and increased levels of T cell population (Figures 4C, 5C) were seen in the presence of old age donor-derived OCs.

### Osteoclasts from older individuals induced lower levels of activation in NK cells

**Figure 6:**
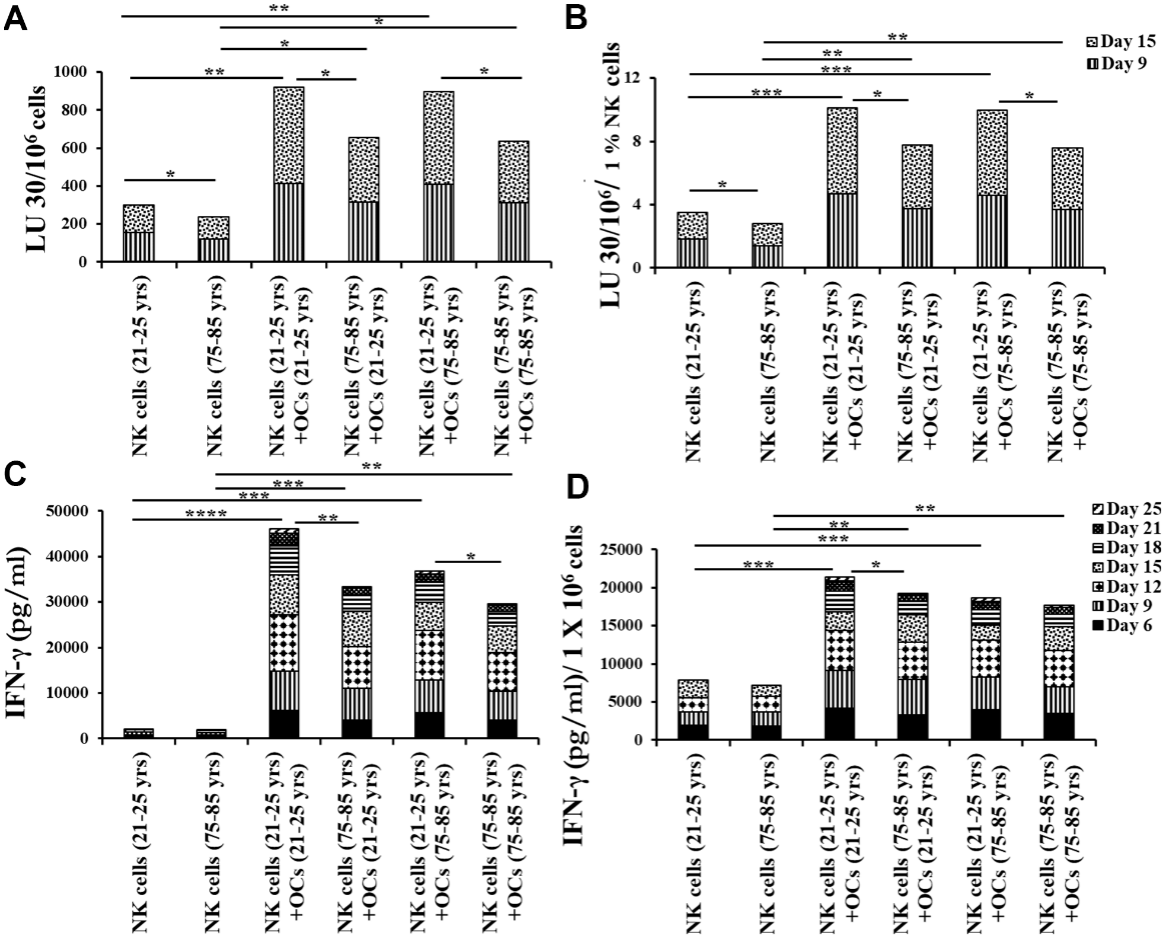
**Older individual-derived OCs induced lower levels of activation in NK cells.** Osteoclasts (OCs) were generated as described in the Materials and Methods section. NK cells and OCs co-culture was performed as described in Figures 2, 5. NK cell-mediated cytotoxicity against OSCSCs was determined on days 9 and 15 using a standard 4-hour ^51^Cr release assay. The lytic units 30/10^6^ cells were determined using the inverse number of NK cells required to lyse 30% of OSCSCs x 100 (**A**). Lytic units per 1 % NK cells were determined based on the percentages of CD16+CD56+ NK cells in the cultures obtained by flow cytometric analysis (**B**). The supernatants were harvested from the cultures on days 6, 9, 12, 15, 18, 21, and 25 to determine IFN-γ secretion using single ELISA (**C**), and the levels IFN-γ pg/ml were adjusted based on per million of cells (**D**). ****(p-value <0.0001), ***(*p*-value 0.0001-0.001).

Next, we compared NK cell-mediated cytotoxicity and secretion level of IFN-y in IL-2+anti-CD16 mAbs treated NK cells co-cultured with OCs either from young or old age donors in the presence of probiotic bacteria. NK cells and OCs from young and old age donors were co-cultured in criss-cross manner. NK cells both from young-age and old-age donors expressed lower levels of NK cell-mediated cytotoxicity (Figure 6A, 6B and Supplementary Figures 4A, 4B, 5A, 5B) and decreased secretion levels of IFN-γ (Figure 6C, 6D and Supplementary Figures 4C, 4D, 5C, 5D) in the presence of old age donor-derived OCs.

## DISCUSSION

We have long been aware of the decrease in NK cell function in elderly individuals [8, 14–16], however, the details of such a decrease have not been investigated clearly. In this paper, we present a number of factors that distinguish the status of NK cells from young and old individuals. Such differences may be the cornerstone of many diseases including cancer which is more prevalent in older individuals than the young population. In addition, this study may reveal potential targets that can be used in order to improve or even reverse the deficiencies seen in the NK cells of older individuals. As shown in other studies [8, 14–16] we also show that there are significant differences in NK function between young and aged individuals (Figure 1B, 1C). In addition, the levels of OC-induced NK expansion in older individuals are much less due to the faster expansion of T cells. This is similar to what we saw with the NK cells from cancer patients [36]. In accordance with a lower function of NK cells in aged individuals we also see a decrease in the activation markers on NK cells from aged individuals, similar to those seen in cancer patients [36] (Figure 1A). We saw a significant drop in the cytotoxic function of NK cells after expansion from aged individuals, however, this did not completely correlate with the decrease in IFN-γ secretion (Figure 3). The level of decrease in the IFN-γ secretion on average was less in aged individuals when compared to those of the cytotoxic functions. These differences could be that the cytotoxicity is very specific to NK targets whereas IFN-γ secretion can also be triggered by the T cells.

We have previously established the role of osteoclasts (OCs) in the greater expansion of NK cells coined as supercharged NK cells [36]. To understand whether the OCs from either young or old donors have differential effects on the expansion of NK cells, we assessed the phenotype and function of OCs and studied the potential contribution of these cells to the decrease in NK function of aged individuals as compared to young people. In general, we observed decreases in different surface markers of OCs in aged individuals when compared to young individuals (Supplementary Figure 3). When the levels and function of NK cells were assessed between the OCs from the aged individuals vs. young individuals with the NK cells obtained from young people, there was a decrease on average in the numbers of NK cells in samples containing the old individuals' OCs with the young donors’ NK cells, however, this decrease was not substantial (Figures 4, 5). Similar trends were observed when NK cells from old individuals were cultured either with young or old individuals’ OCs. In contrast, NK cells from young or old individuals cultured with different OCs substantially decreased NK cell numbers. Thus, these experiments show that NK cells contribute to the defect more than the OCs from young and old individuals. Similar trends were observed when IFN-γ secretion was considered in the co-cultures of the same NK cells with OCs from the young and old individuals, however, such differences were not obvious when cytotoxicity was considered (Figure 6 and Supplementary Figures 4, 5). Again, the differences in cytotoxicity and IFN-γ secretion were substantial when different NK cells (either from young or old) were considered with OCs, indicating that NK cells contribute to the defect more than OCs. These experiments are very complex; however, they indicate that any kind of treatment or intervention should be at the level of NK cells. Therefore, NK cells from young individuals when given to older individuals may substantially improve the NK function in the older individuals. Such experiments should wait for future investigations. When designing effective therapeutic strategies for the treatment of cancer, in an allogeneic system, NK cells from young individuals should yield much better results when cultured with young osteoclasts when compared to those from older individuals. It also appears that as individuals age, the levels of different surface receptors both on NK cells and OCs diminish, likely contributing to the decline of NK cell numbers and function. At present, it is unclear why the NK cells and OCs decrease the levels of important receptors and ligands on the surface of cells from old individuals, and what could be done to restore their levels. Strategies to prevent the loss of receptors or restore the levels utilizing genetic engineering, blocking inhibitory receptors or immunotherapeutics such as checkpoint inhibitors and NK cell therapy etc. should be effective in restoring the numbers and the function of NK cells in older individuals, and likely prevent the initiation and progression of cancer and other chronic diseases.

For a long time, it was speculated that NK cells were important for longevity. The optimal function of NK cells plays a key role in protecting the elderly population from infections, cancer, autoimmune diseases, and neurogenerative diseases. Reduced NK cell function at older age contributes to the onset or spread of these old age-related health problems [8, 14–16]. Alzheimer’s disease, several cancers, inflammatory diseases like arthritis, bone disorders like osteoporosis, cardiovascular issues, atherosclerosis, eye diseases, and age-related macular degeneration are common after 65 years of age [43]. NK cells are important immune cells to fight against these age-related illnesses, and they directly play a crucial role in activating adaptive immune cells ultimately maintaining healthy immune system in old age. We now understand why NK cells may have such a crucial role in imparting a long life to individuals. Our recent studies demonstrated that infusion of supercharged NK cells to older individuals was able to increase and maintain the percentages of NK cells in the peripheral blood for an extended period of time (manuscript in prep). Therefore, sNK cells have the ability to reverse inactivation and loss of autologous NK cells in older age individuals, by offering a therapeutic potential to decrease disease induction in older age individuals.

## MATERIALS AND METHODS

### Cell lines, reagents, and antibodies

Oral squamous carcinoma stem cells (OSCSCs) were isolated from patients with tongue tumors at UCLA, and are used as NK cell target to determine NK cell-mediated cytotoxicity [4, 44–46]. NK cells and OSCSCs were cultured in RPMI 1640 (Invitrogen by Life Technologies, Carlsbad, CA, USA), supplemented with 10% fetal bovine serum (FBS) (Gemini Bio-Products, West Sacramento, CA, USA). Recombinant IL-2 was obtained from NIH-BRB. Anti-CD16 mAbs, antibodies used for flow cytometer, and ELISA kits for IFN-γ were purchased from Biolegend (San Diego, CA, USA). Chromium-51 was purchased from PeproTech (Cranbury, NJ, USA).

### Purification of human NK cells and monocytes

Written informed consents, approved by UCLA Institutional Review Board (IRB), were obtained from healthy individuals, and all procedures were approved by the UCLA-IRB. Peripheral blood was separated using ficoll-hypaque centrifugation, after which the white, cloudy layer, containing peripheral blood mononuclear cells (PBMCs) was harvested. NK cells and monocytes were negatively selected from PBMCs using the EasySep® Human NK cell enrichment and EasySep® Human monocytes enrichments kits, respectively, purchased from Stem Cell Technologies (Vancouver, BC, Canada). Purified NK cells and monocytes were stained with anti-CD16 and anti-CD14, respectively, to measure purity using flow cytometric analysis. Samples showing greater than 95% purity were used for the study.

### Generation of osteoclasts and expansion of NK cells

To generate osteoclasts (OCs), monocytes were cultured in alpha-MEM media supplemented with M-CSF (25 ng/mL) and RANKL (25 ng/mL) for 21 days, media was replenished every three days. Human purified NK cells were activated with rh-IL-2 (1000 U/ml) and anti-CD16 mAb (3 μg/ml) for 18-20 hours before they were co-cultured with OCs and probiotic bacteria sAJ2 (OCs:NK:sAJ2; 1:2:4) in RPMI 1640 medium containing 10% FBS. Probiotic bacteria, AJ2 is a combination of eight different strains of gram-positive probiotic bacteria (*Streptococcus thermophiles, Bifidobacterium longum, Bifidobacterium breve, Bifidobacterium infantis, Lactobacillus acidophilus, Lactobacillus plantarum, Lactobacillus paracasei and Lactobacillus bulgaricus*) elected for their superior ability to induce optimal secretion of both pro-inflammatory and anti-inflammatory cytokines in NK cells [4]. The medium was refreshed every three days with RPMI containing rh-IL-2 (1500 U/ml).

### Surface staining

Staining was performed by labeling the cells with antibodies as described previously [47–49]. Flow cytometric analysis was performed using Beckman Coulter Epics XL cytometer (Brea, CA, USA) and results were analyzed in the FlowJo vX software (Ashland, OR, USA).

### Enzyme-linked immunosorbent assays (ELISAs)

Single ELISAs were performed as previously described [49]. To analyze and obtain the cytokine and chemokine concentration, a standard curve was generated by either two- or three-fold dilutions of recombinant cytokines provided by the manufacturer.

### ^51^Cr release cytotoxicity assay

The ^51^Cr release cytotoxicity assay was performed as previously described [50]. Briefly, different ratios effectors (NK cells) and ^51^Cr–labeled target cells (OSCSCs) were incubated for four hours. After this, the supernatants were harvested from each sample, and the released radioactivity was counted using the gamma counter. The percentage specific cytotoxicity was calculated as follows:


$$\%\text{cytotoxicity}=\frac{\text{Experimental cpm}-\text{spontaneous cpm}}{\text{Total cpm}-\text{spontaneous cpm}}$$

Lytic units (LU) 30/10^6^ are calculated by using the inverse of the number of NK cells needed to lyse 30% of OSCSCs ×100.

### Statistical analysis

Prism-9 software was used for statistical analysis. An unpaired or paired, two-tailed Student’s t-test was performed for experiments with two groups. One-way ANOVA with a Bonferroni post-test was used to compare different groups for experiments with more than two groups. Duplicate or triplicate samples were used for assessment, and “n” donates the number of donors or samples used. The following symbols represent the levels of statistical significance within each analysis: ****(p-value <0.0001), ***(*p*-value 0.0001-0.001), **(*p*-value 0.001-0.01), *(*p*-value 0.01-0.05).

## Supplementary Material

Supplementary Figures

Supplementary Tables
