# Supplementary material for: Decreased surface receptors, function, and suboptimal osteoclasts-induced cell expansion in natural killer (NK) cells of elderly subjects
Source: Aging (Albany NY). 2025 Mar 26;17(3):798–821. doi: 10.18632/aging.206226 (PMC11984426; doi:10.18632/aging.206226)
Supplement: Supplementary Figures [file aging-17-206226-s001.pdf]

## SUPPLEMENTARY FIGURES

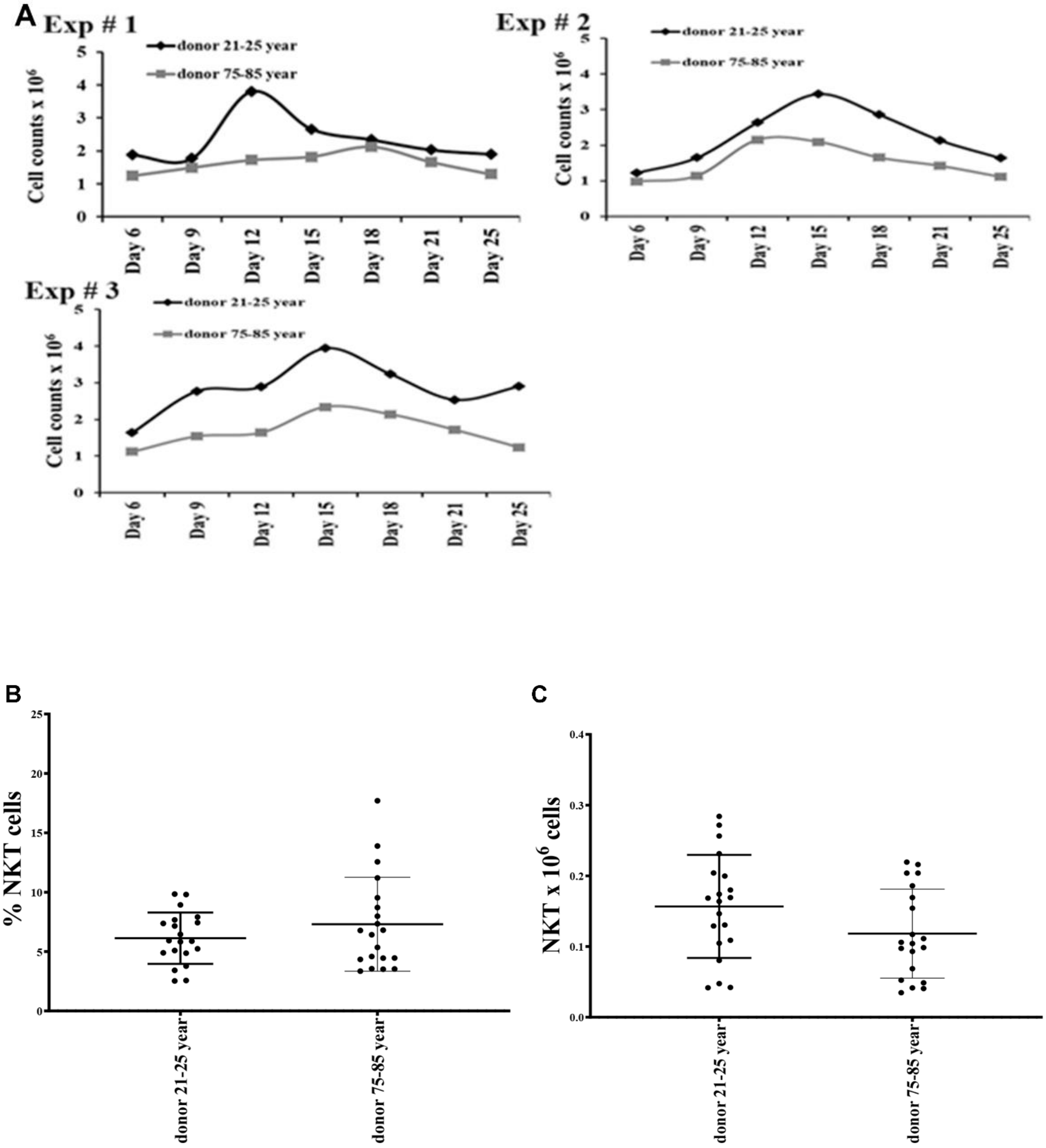

D

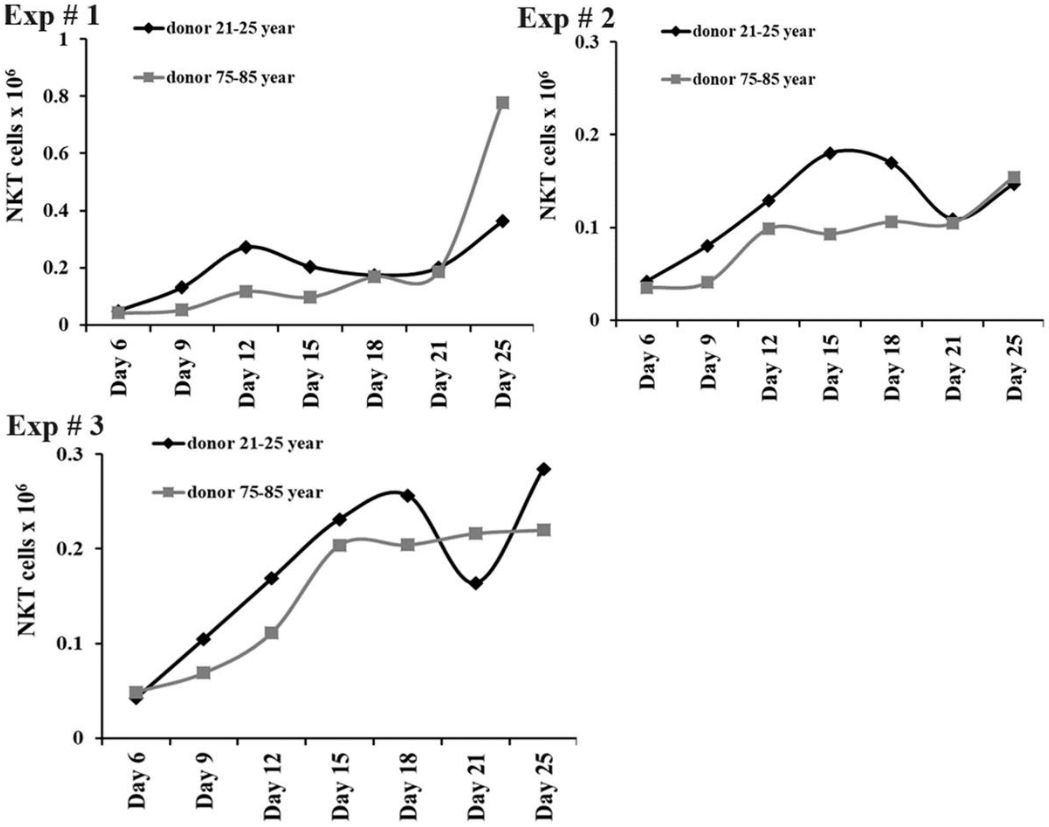
Exp # 1
Exp # 2
Exp # 3
donor 21-25 year
donor 75-85 year
NKT cells x 10^6
Day 6
Day 9
Day 12
Day 15
Day 18
Day 21
Day 25

E

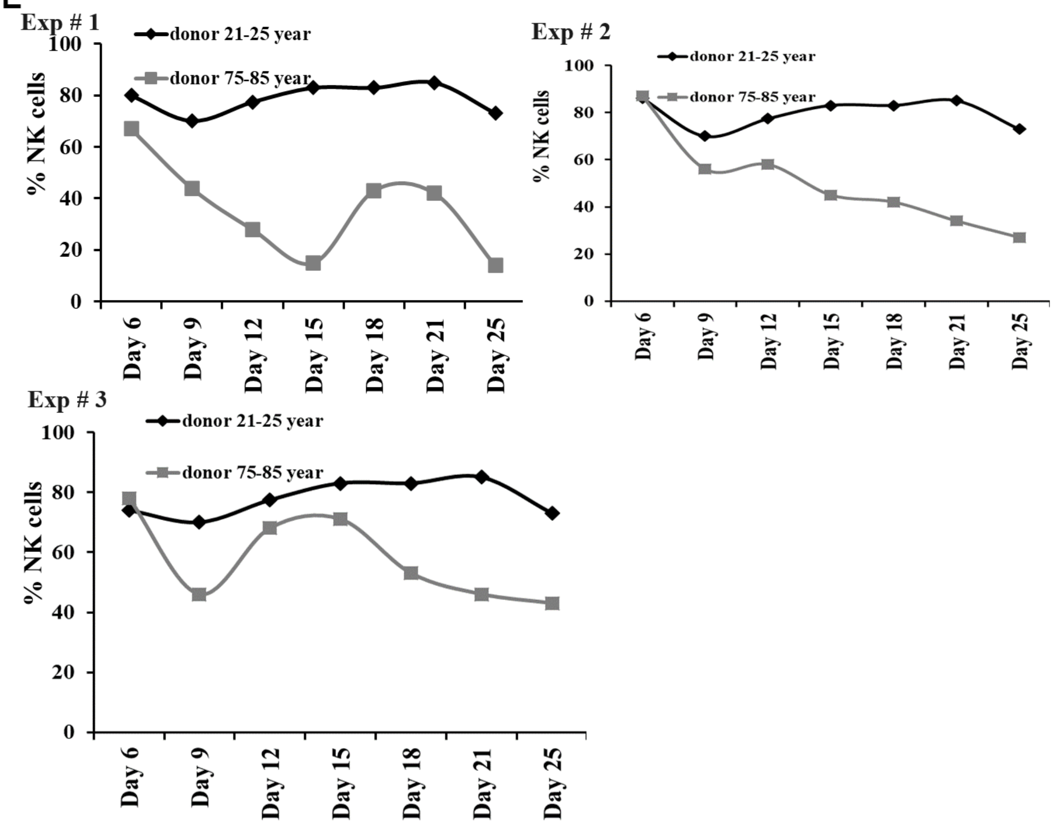
Exp # 1
Exp # 2
Exp # 3
donor 21-25 year
donor 75-85 year
% NK cells
Day 6
Day 9
Day 12
Day 15
Day 18
Day 21
Day 25

F

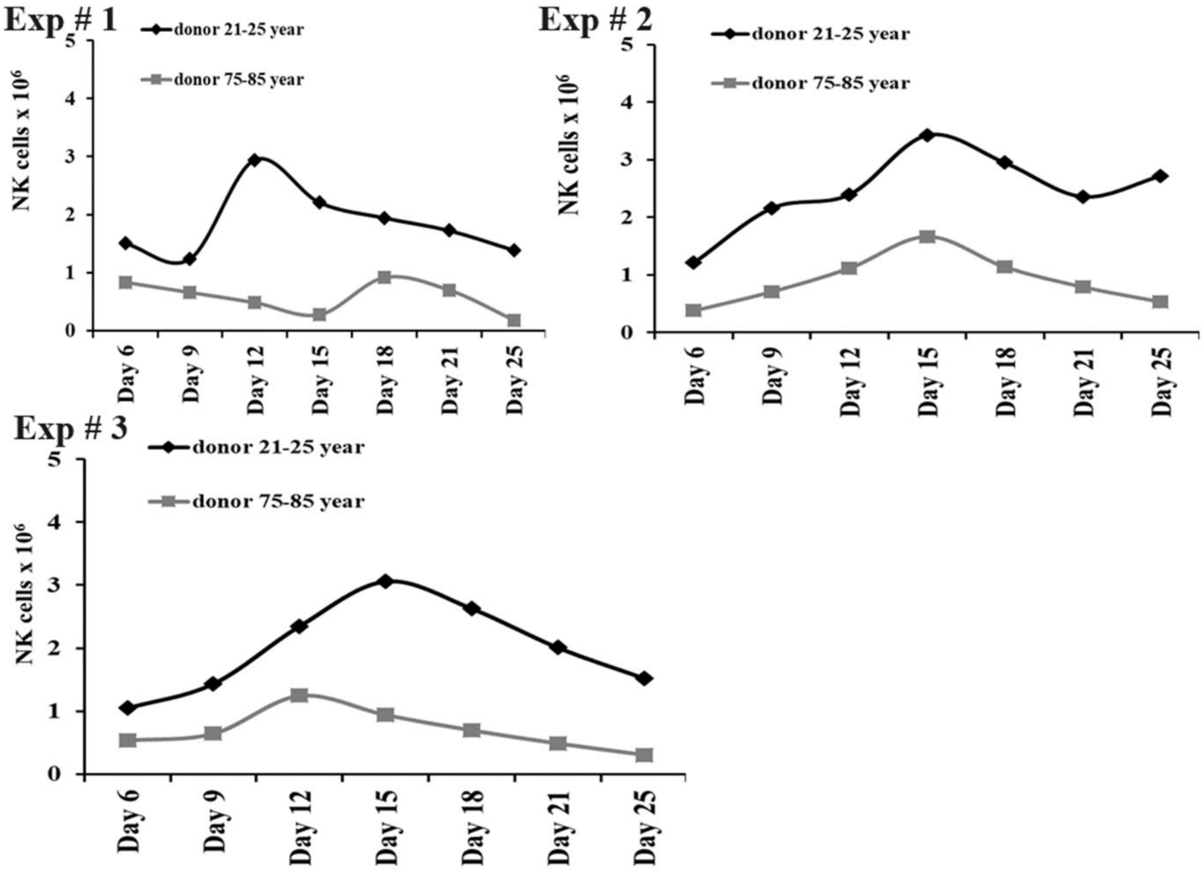
Exp # 1
donor 21-25 year
donor 75-85 year
NK cells x $10^6$
0
1
2
3
4
5
Day 6
Day 9
Day 12
Day 15
Day 18
Day 21
Day 25
Exp # 2
donor 21-25 year
donor 75-85 year
NK cells x $10^6$
Exp # 3
donor 21-25 year
donor 75-85 year
NK cells x $10^6$

G

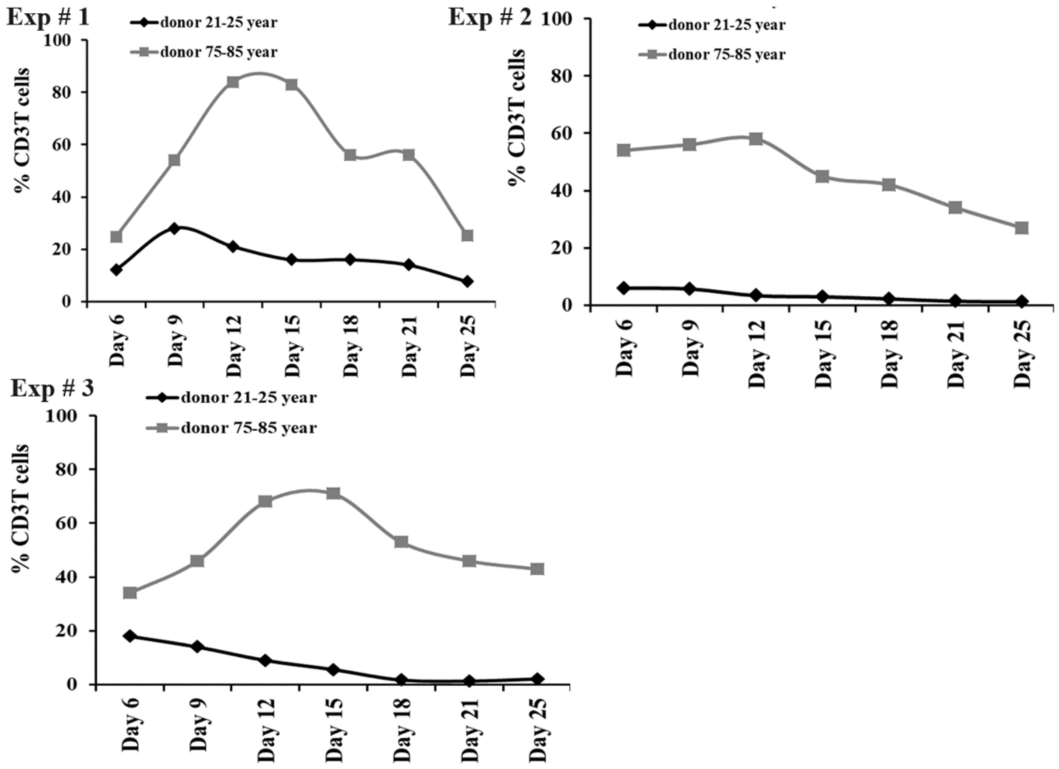
Exp # 1
donor 21-25 year
donor 75-85 year
% CD3T cells
0
20
40
60
80
100
Day 6
Day 9
Day 12
Day 15
Day 18
Day 21
Day 25
Exp # 2
donor 21-25 year
donor 75-85 year
% CD3T cells
Exp # 3
donor 21-25 year
donor 75-85 year
% CD3T cells

H

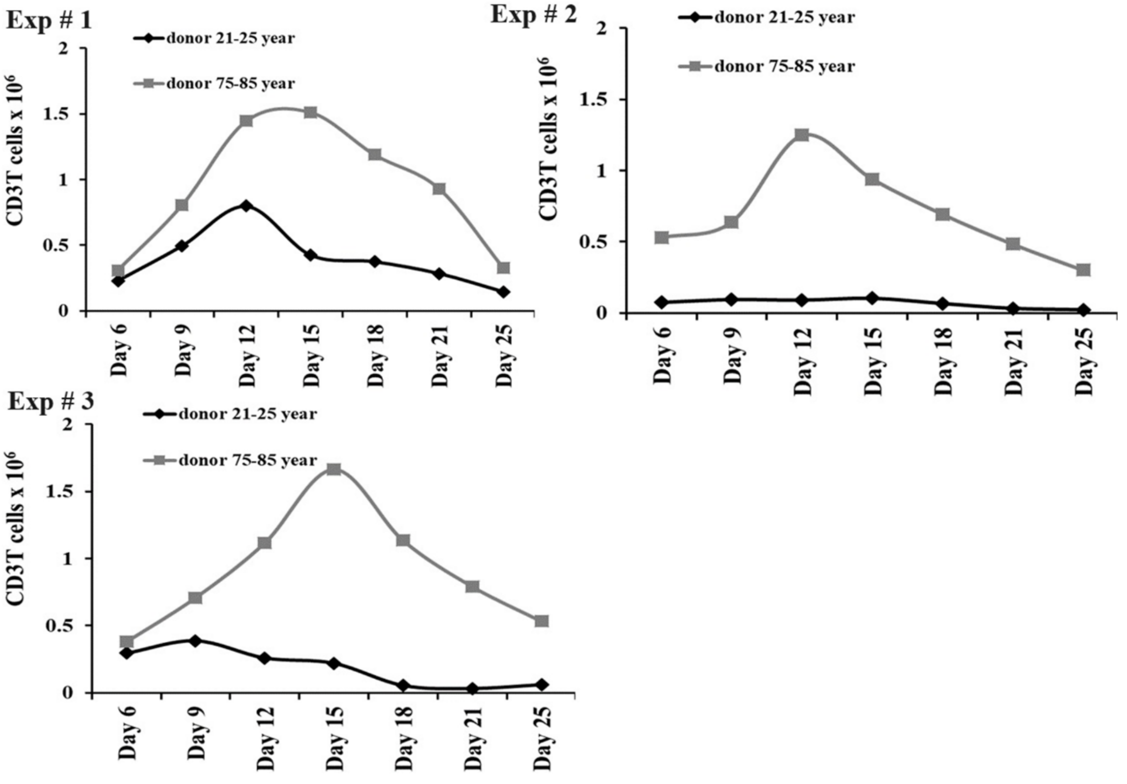
Exp # 1
Exp # 2
Exp # 3
donor 21-25 year
donor 75-85 year
CD3T cells x 10^6
0
0.5
1
1.5
2
Day 6
Day 9
Day 12
Day 15
Day 18
Day 21
Day 25

I

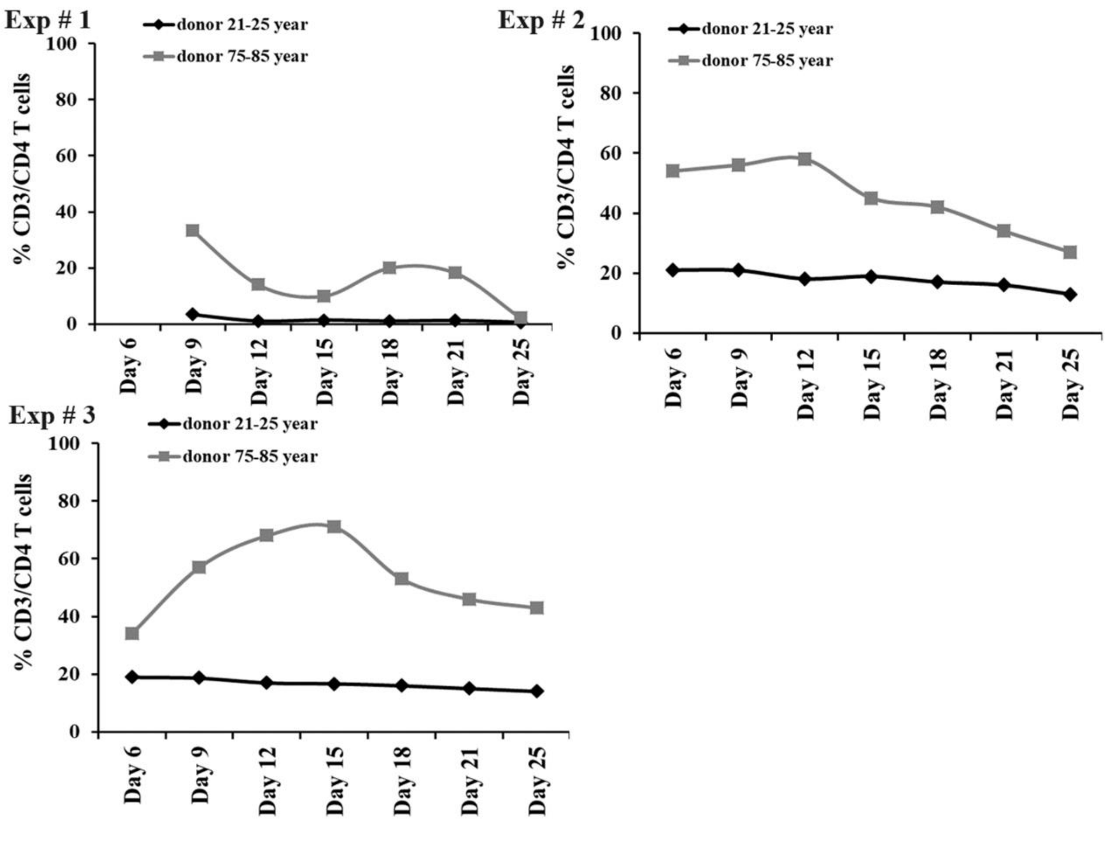
Exp # 1
Exp # 2
Exp # 3
donor 21-25 year
donor 75-85 year
% CD3/CD4 T cells
0
20
40
60
80
100
Day 6
Day 9
Day 12
Day 15
Day 18
Day 21
Day 25

J

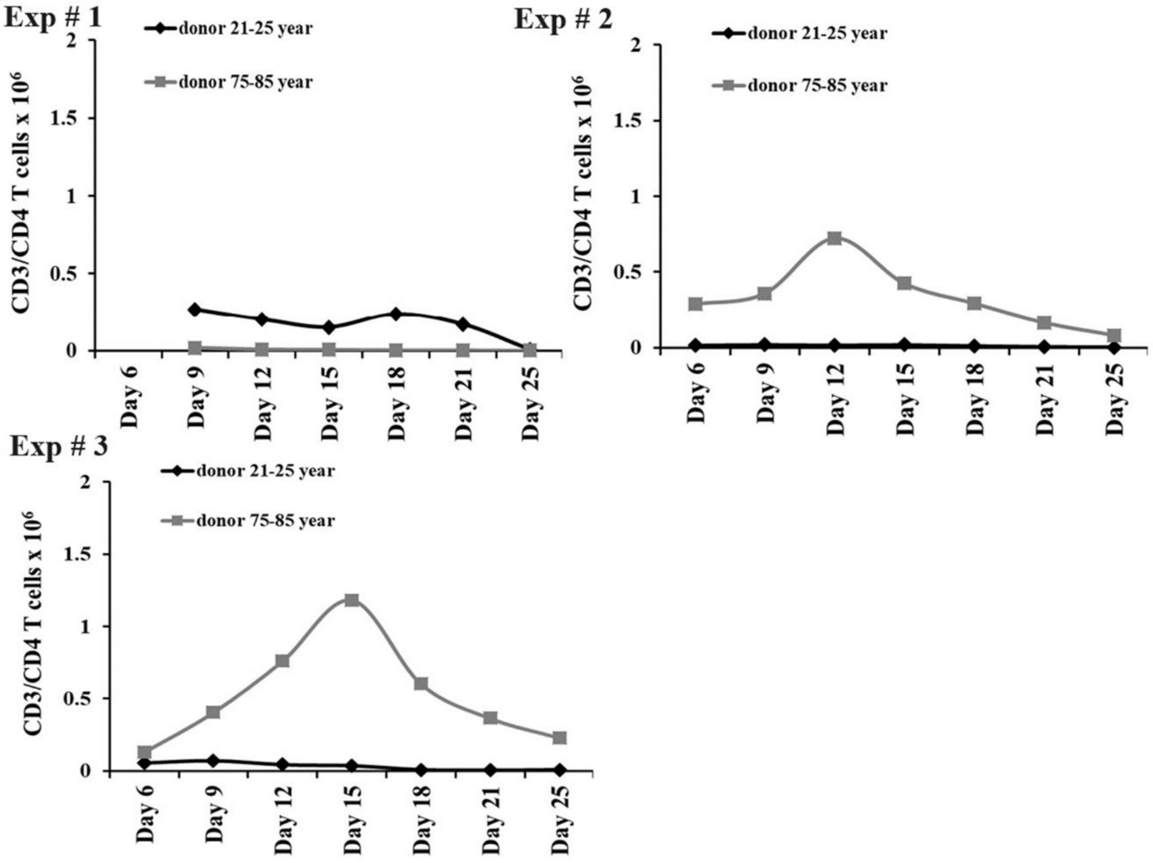
Exp # 1
Exp # 2
Exp # 3
donor 21-25 year
donor 75-85 year
CD3/CD4 T cells x 10⁶
0
0.5
1
1.5
2
Day 6
Day 9
Day 12
Day 15
Day 18
Day 21
Day 25

K

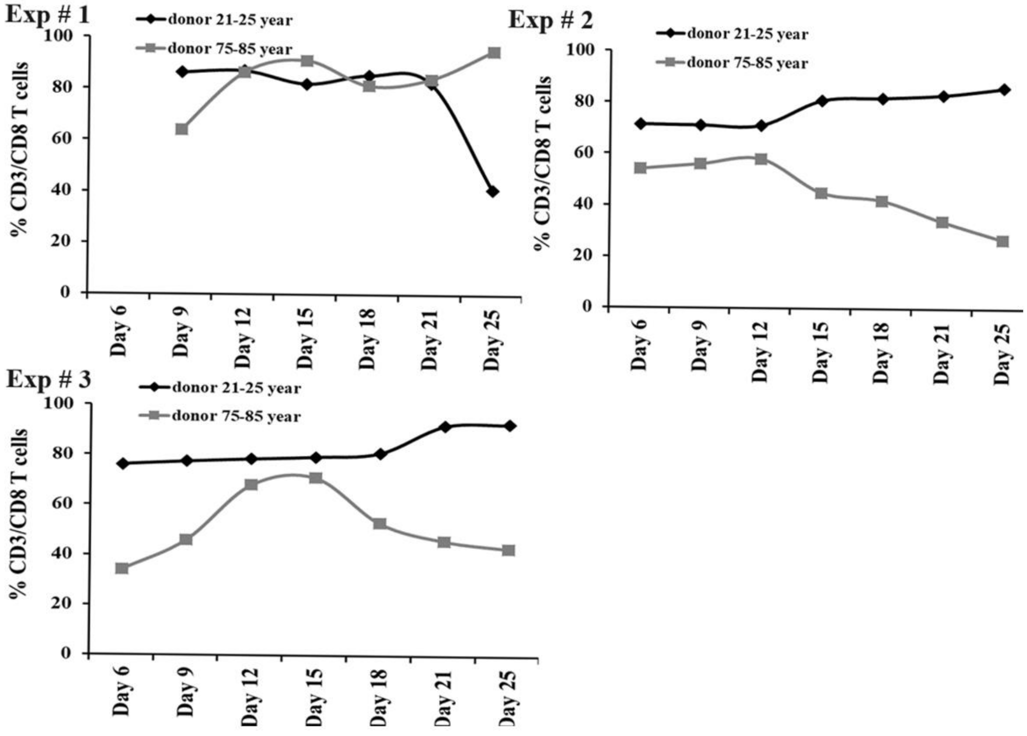
Exp # 1
Exp # 2
Exp # 3
donor 21-25 year
donor 75-85 year
% CD3/CD8 T cells
0
20
40
60
80
100
Day 6
Day 9
Day 12
Day 15
Day 18
Day 21
Day 25

L

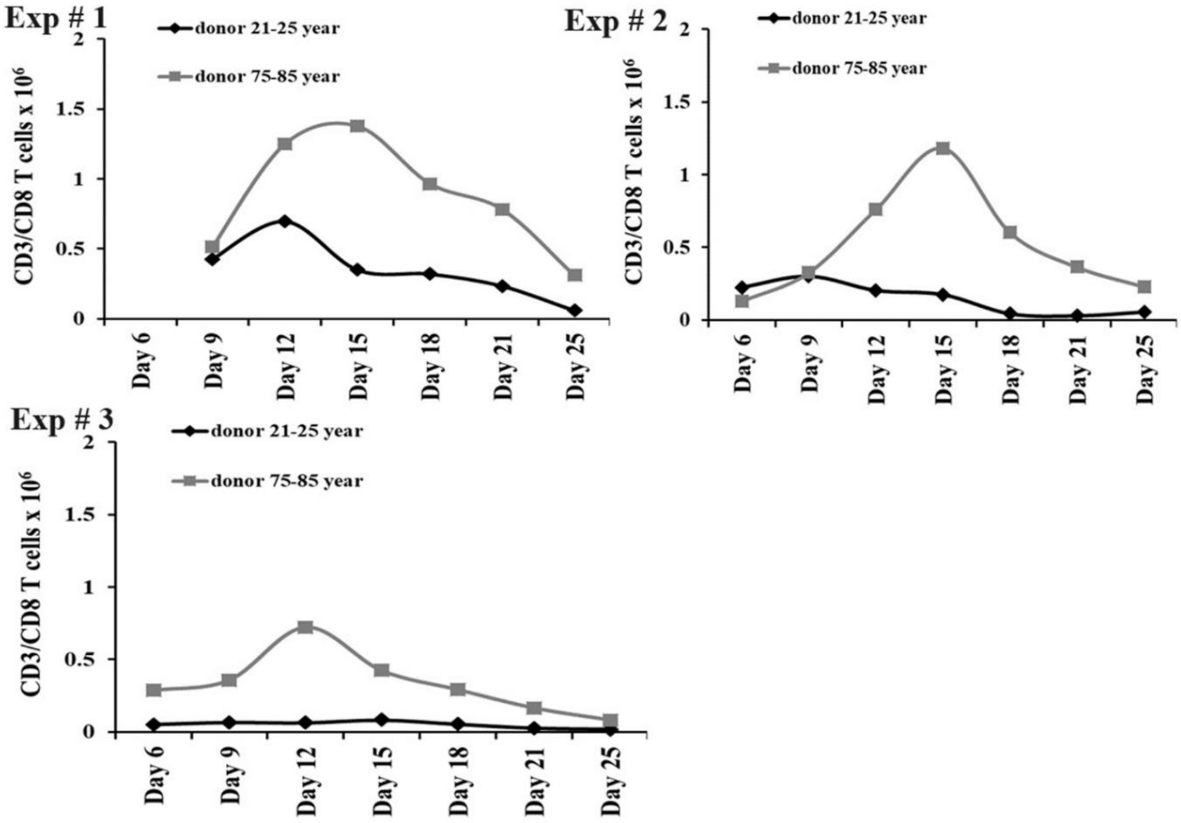


**Supplementary Figure 1. OCs induced lower levels of cell expansion in old-age donor NK cells.** Osteoclasts (OCs) were generated as described in the Materials and Methods section. NK cells (0.5x$10^6$ cells/ 2ml) were a combination of IL-2 (1000 U/ml) and anti-CD16mAb (3µg/ml) for 18 hours before they were co-cultured with OCs and sAJ2 (1:2:4: OCs:NK:sAJ2). NK cells were counted on days 6, 9, 12, 15, 18, 21, and 25, day 0 counts were 0.5x$10^6$ cells/2 ml, and 0.5x$10^6$ cells/2 ml were cultured every 3 days (**A**). CD3+CD16+CD56+ NKT cells, and the number of NKT cells (**B**–**D**), CD16+CD56+ NK cells and the number of NK cells (**E**, **F**), CD3+ T cells and the number of T cells (**G**, **H**), CD3+CD4+ T cells and the number of CD4+ T cells (**I**, **J**), CD3+CD8+ T cells and the number of CD8+ T cells (**K**, **L**), were determined counted on days 6, 9, 12, 15, 18, 21, and 25 in expanding cells.

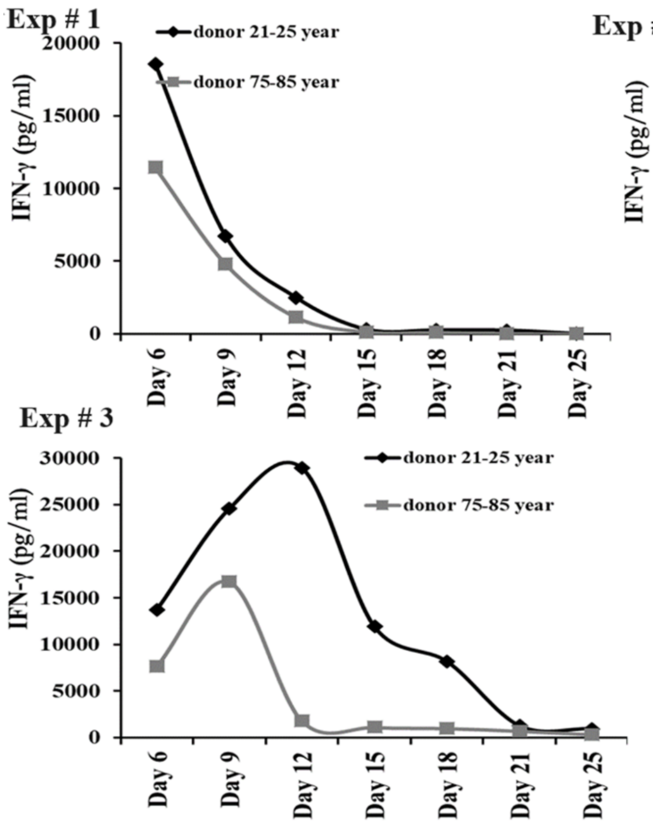


**Supplementary Figure 2. OCs induced lower levels of increased IFN-γ secretion in old-age donor NK cells.** Osteoclasts (OCs) were generated as described in the Materials and Methods section. NK cells and OCs co-culture was performed as described in Supplementary Figure 1. The supernatants were harvested from the cultures on days 6, 9, 12, 15, 18, 21, and 25 to determine IFN-γ secretion using single ELISA.

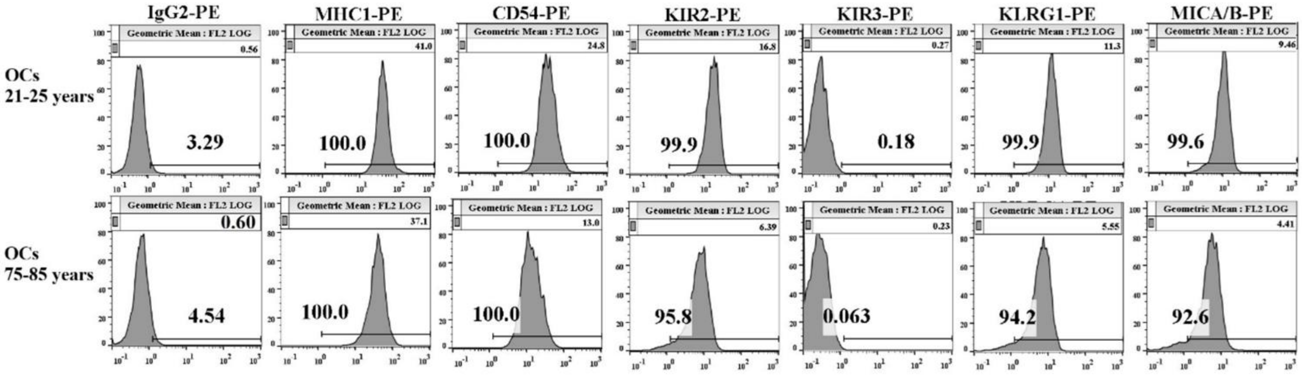


**Supplementary Figure 3. Reduced surface receptor expressions on old age Ocs.** Osteoclasts (OCs) were generated as described in the Materials and Methods section. The surface markers MHC-class I, CD54, KIR2, KIR3, KLRG1, and MICA/B were determined on OCs using flow cytometric analysis (one of two representative experiments is shown in the figure).

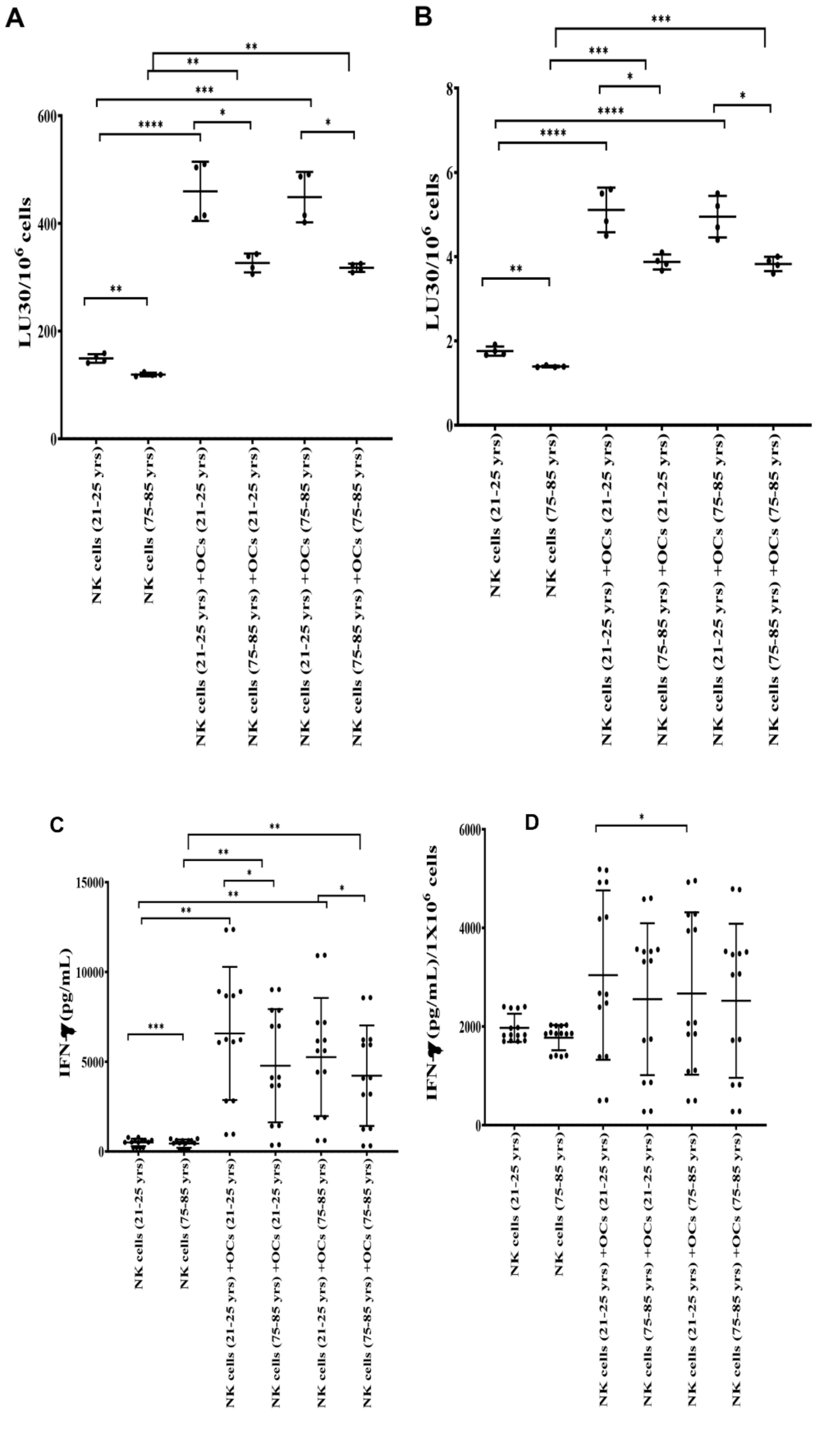


**Supplementary Figure 4. Old-age donor-derived OCs induced lower levels of activation in NK cells.** Osteoclasts (OCs) were generated as described in the Materials and Methods section. NK cells (0.5x10$^6$ cells/ 2ml) were a combination of IL-2 (1000 U/ml) and anti-CD16mAb (3µg/ml) for 18 hours before they were co-cultured with criss-cross OCs and sAJ2 (1:2:4: OCs:NK:sAJ2). NK cell-mediated cytotoxicity against OSCSCs was determined on days 9 and 15 using a standard 4-hour $^{51}$Cr release assay. The lytic units 30/10$^6$ cells were determined using the inverse number of NK cells required to lyse 30% of OSCSCs x 100 (**A**). Lytic units per 1 % NK cells were determined based on the percentages of CD16+/CD56+ NK cells in the cultures obtained by flow cytometric analysis (**B**). The supernatants were harvested from the cultures on days 6, 9, 12, 15, 18, 21, and 25 to determine IFN-γ secretion using single ELISA (**C**), and the levels were adjusted based on per million of cells (**D**). ****(p-value <0.0001), ***(*p*-value 0.0001-0.001).

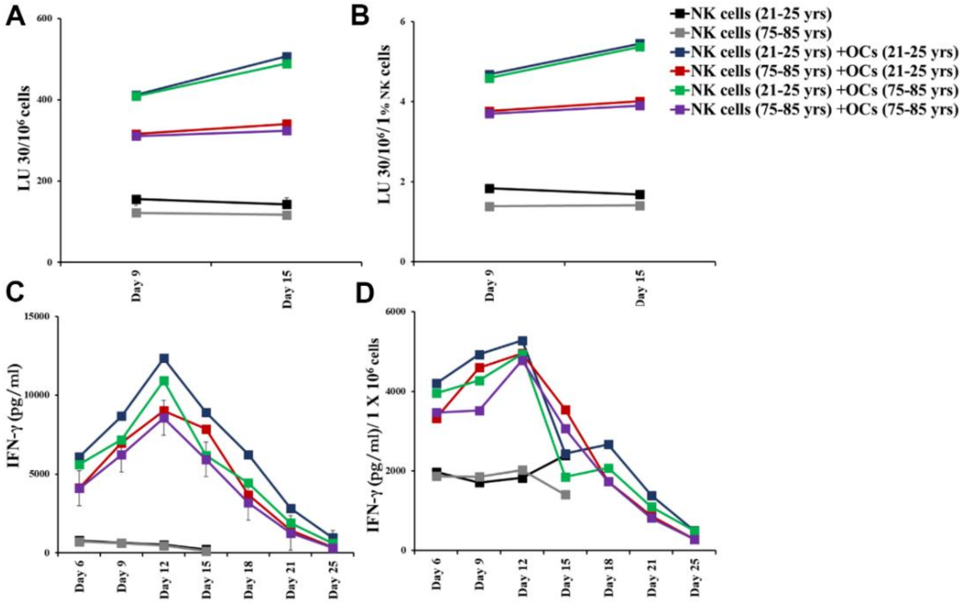


**P-values of Supplementary Figure 5**

A

| P values | Day 9 | Day 15 |
|---|---|---|
| NK (21-25 y) vs. NK(75-85 y) | * | * |
| NK (21-25 y) vs. NK(21-25 y)+OCs(21-25 y) | * | * |
| NK (21-25 y) vs. NK(75-85 y)+OCs(21-25 y) | * | **** |
| NK (21-25 y) vs. NK(21-25 y)+OCs(75-85 y) | * | **** |
| NK (21-25 y) vs. NK(75-85 y)+OCs(75-85 y) | * | * |
| NK (75-85 y) vs. NK(21-25 y)+OCs(21-25 y) | * | * |
| NK (75-85 y) vs. NK(75-85 y)+OCs(21-25 y) | * | * |
| NK (75-85 y) vs. NK(21-25 y)+OCs(75-85 y) | * | * |
| NK (75-85 y) vs. NK(75-85 y)+OCs(75-85 y) | * | * |
| NK(21-25 y)+OCs(21-25 y) vs. NK (75-85 y)+OCs(21-25 y) | * | * |
| NK(21-25 y)+OCs(21-25 y) vs. NK (21-25 y)+OCs(75-85 y) | ns | ns |
| NK(21-25 y)+OCs(21-25 y) vs. NK (75-85 y)+OCs(75-85 y) | * | * |
| NK(75-85 y)+OCs(21-25 y) vs. NK (21-25 y)+OCs(75-85 y) | * | **** |
| NK(75-85 y)+OCs(21-25 y) vs. NK (75-85 y)+OCs(75-85 y) | ns | ns |
| NK(21-25 y)+OCs(75-85 y) vs. NK (75-85 y)+OCs(75-85 y) | ns | * |

B

| P values | Day 9 | Day 15 |
|---|---|---|
| NK (21-25 y) vs. NK(75-85 y) | ns | * |
| NK (21-25 y) vs. NK(21-25 y)+OCs(21-25 y) | ns | * |
| NK (21-25 y) vs. NK(75-85 y)+OCs(21-25 y) | * | ns |
| NK (21-25 y) vs. NK(21-25 y)+OCs(75-85 y) | ns | ns |
| NK (21-25 y) vs. NK(75-85 y)+OCs(75-85 y) | ns | ns |
| NK (75-85 y) vs. NK(21-25 y)+OCs(21-25 y) | ns | * |
| NK (75-85 y) vs. NK(75-85 y)+OCs(21-25 y) | * | * |
| NK (75-85 y) vs. NK(21-25 y)+OCs(75-85 y) | ns | * |
| NK (75-85 y) vs. NK(75-85 y)+OCs(75-85 y) | ns | ns |
| NK(21-25 y)+OCs(21-25 y) vs. NK (75-85 y)+OCs(21-25 y) | ns | * |
| NK(21-25 y)+OCs(21-25 y) vs. NK (21-25 y)+OCs(75-85 y) | ns | ns |
| NK(21-25 y)+OCs(21-25 y) vs. NK (75-85 y)+OCs(75-85 y) | * | ns |
| NK(75-85 y)+OCs(21-25 y) vs. NK (21-25 y)+OCs(75-85 y) | ns | * |
| NK(75-85 y)+OCs(21-25 y) vs. NK (75-85 y)+OCs(75-85 y) | ns | ns |
| NK(21-25 y)+OCs(75-85 y) vs. NK (75-85 y)+OCs(75-85 y) | **** | ns |

C

| P values | Day 6 | Day 9 | Day 12 | Day 15 | Day 18 | Day 21 | Day 25 |
|---|---|---|---|---|---|---|---|
| NK (21-25 y) vs. NK(75-85 y) | * | ns | ns | ns | | | |
| NK (21-25 y) vs. NK(21-25 y)+OCs(21-25 y) | * | * | * | * | | | |
| NK (21-25 y) vs. NK(75-85 y)+OCs(21-25 y) | * | * | * | * | | | |
| NK (21-25 y) vs. NK(21-25 y)+OCs(75-85 y) | * | * | * | * | | | |
| NK (21-25 y) vs. NK(75-85 y)+OCs(75-85 y) | * | * | * | * | | | |
| NK (75-85 y) vs. NK(21-25 y)+OCs(21-25 y) | * | * | * | * | | | |
| NK (75-85 y) vs. NK(75-85 y)+OCs(21-25 y) | * | * | * | * | | | |
| NK (75-85 y) vs. NK(21-25 y)+OCs(75-85 y) | * | * | * | * | | | |
| NK (75-85 y) vs. NK(75-85 y)+OCs(75-85 y) | * | * | * | * | | | |
| NK(21-25 y)+OCs(21-25 y) vs. NK (75-85 y)+OCs(21-25 y) | * | * | * | * | * | * | * |
| NK(21-25 y)+OCs(21-25 y) vs. NK (21-25 y)+OCs(75-85 y) | * | * | * | * | * | * | * |
| NK(21-25 y)+OCs(21-25 y) vs. NK (75-85 y)+OCs(75-85 y) | * | * | * | * | * | * | * |
| NK(75-85 y)+OCs(21-25 y) vs. NK (21-25 y)+OCs(75-85 y) | * | * | * | * | * | ns | ns |
| NK(75-85 y)+OCs(21-25 y) vs. NK (75-85 y)+OCs(75-85 y) | ns | * | * | * | * | ns | ns |
| NK(21-25 y)+OCs(75-85 y) vs. NK (75-85 y)+OCs(75-85 y) | * | * | * | ns | * | ns | * |

D

| P values | Day 6 | Day 9 | Day 12 | Day 15 | Day 18 | Day 21 | Day 25 |
|---|---|---|---|---|---|---|---|
| NK (21-25 y) vs. NK(75-85 y) | ns | * | **** | * | | | |
| NK (21-25 y) vs. NK(21-25 y)+OCs(21-25 y) | * | * | * | ns | | | |
| NK (21-25 y) vs. NK(75-85 y)+OCs(21-25 y) | * | * | * | * | | | |
| NK (21-25 y) vs. NK(21-25 y)+OCs(75-85 y) | * | * | * | * | | | |
| NK (21-25 y) vs. NK(75-85 y)+OCs(75-85 y) | * | * | * | * | | | |
| NK (75-85 y) vs. NK(21-25 y)+OCs(21-25 y) | * | * | * | * | | | |
| NK (75-85 y) vs. NK(75-85 y)+OCs(21-25 y) | * | * | * | * | | | |
| NK (75-85 y) vs. NK(21-25 y)+OCs(75-85 y) | **** | * | * | * | | | |
| NK (75-85 y) vs. NK(75-85 y)+OCs(75-85 y) | * | * | * | * | | | |
| NK(21-25 y)+OCs(21-25 y) vs. NK (75-85 y)+OCs(21-25 y) | * | * | * | * | * | * | * |
| NK(21-25 y)+OCs(21-25 y) vs. NK (21-25 y)+OCs(75-85 y) | * | * | * | ns | * | * | ns |
| NK(21-25 y)+OCs(21-25 y) vs. NK (75-85 y)+OCs(75-85 y) | * | * | * | ns | * | **** | * |
| NK(75-85 y)+OCs(21-25 y) vs. NK (21-25 y)+OCs(75-85 y) | * | * | * | * | * | * | * |
| NK(75-85 y)+OCs(21-25 y) vs. NK (75-85 y)+OCs(75-85 y) | * | * | * | * | ns | * | ns |
| NK(21-25 y)+OCs(75-85 y) vs. NK (75-85 y)+OCs(75-85 y) | * | * | ns | * | * | * | * |

**Supplementary Figure 5. Old-age donor-derived OCs induced lower levels of activation in NK cells.** Osteoclasts (OCs) were generated as described in the Materials and Methods section. NK cells and OCs co-culture was performed as described in Fig. S4. NK cell-mediated cytotoxicity against OSCSCs was determined on days 9 and 15 using a standard 4-hour $^{51}$Cr release assay. The lytic units 30/10$^{6}$ cells were determined using the inverse number of NK cells required to lyse 30% of OSCSCs x 100 (**A**). Lytic units per 1 % NK cells were determined based on the percentages of CD16+CD56+ NK cells in the cultures obtained by flow cytometric analysis (**B**). The supernatants were harvested from the cultures on days 6, 9, 12, 15, 18, 21, and 25 to determine IFN-γ secretion using single ELISA (**C**), and the levels IFN-γ pg/ml were adjusted based on per million of cells (**D**). ****(p-value <0.0001), ***(*p*-value 0.0001-0.001).
