# Supplementary material for: Decreased surface receptors, function, and suboptimal osteoclasts-induced cell expansion in natural killer (NK) cells of elderly subjects
Source: Aging (Albany NY). 2025 Mar 26;17(3):798–821. doi: 10.18632/aging.206226 (PMC11984426; doi:10.18632/aging.206226)
Supplement: Supplementary Tables [file aging-17-206226-s002.pdf]

SUPPLEMENTARY TABLES

Supplementary Table 1. Decreased levels of cytotoxicity, and IFN-γ secretions in NK cells from an older donor

Supplementary Table 1A

	Exp # 1		Exp # 2		Exp # 3	
LU 30/10^6 cells	21-25 yrs	75-85 yrs	21-25 yrs	75-85 yrs	21-25 yrs	75-85 yrs
Untreated NK	46	19	34	21	54	31
NK + IL-2	120	76	143	109	133	127
NK + IL-2 + anti-CD16 mAbs	24	19	87	48	63	51

Supplementary Table 1B

	Exp # 1		Exp # 2		Exp # 3	
IFN-γ (pg/ml)	21-25 yrs	75-85 yrs	21-25 yrs	75-85 yrs	21-25 yrs	75-85 yrs
Untreated NK	54	46	48	39	33	61
NK + IL-2	128	97	176	106	159	127
NK + IL-2 + anti-CD16 mAbs	199.88	142.55	302	139	263	181

NK cells were left untreated or were treated with IL-2 (1000 U/ml) or with a combination of IL-2 (1000 U/ml) and anti-CD16 mAbs (3 µg/ml) for 18 hours before they were used as effectors in standard 4-hour ^{51}Cr release assay against OSCSCs **(**n=3, **A)**. The lytic units 30/10^6 cells were determined using the inverse number of NK cells required to lyse 30% of tumors X 100. NK cells left untreated ot treated with IL-2 (1000 U/ml) or with a combination of IL-2 (1000 U/ml) and anti-CD16 mAbs (3 µg/ml) for 18 hours before the supernatants were harvested to determine IFN-γ secretion using single ELISA (n=3, **B)**.

Supplementary Table 2. OCs induced lower levels of cytotoxic activity in old-age donor NK cells

Supplementary Table 2A

	Exp # 1		Exp # 2		Exp # 3	
LU 30/10^6 cells	21-25 yrs	75-85 yrs	21-25 yrs	75-85 yrs	21-25 yrs	75-85 yrs
Day 12	344	42	423	86	412	71
Day 30	357	26	256	38		

Supplementary Table 2B

	Exp # 1		Exp # 2		Exp # 3	
LU 30/10^6 cells/ 1 NK %	21-25 yrs	75-85 yrs	21-25 yrs	75-85 yrs	21-25 yrs	75-85 yrs
Day 12	4.4444444	1.5	4.752809	1.4827586	4.9638554	1.0441176
Day 30	4.890411	1.8571429	2.7526882	1.4074074	na	na

Osteoclasts (OCs) were generated as described in the Materials and Methods section. NK cells and OCs co-culture was performed as described in Figure S1. NK cell-mediated cytotoxicity against OSCSCs was determined on days 9 and 15 using a standard 4-hour ^{51}Cr release assay. The lytic units 30/10^6 cells were determined using the inverse number of NK cells required to lyse 30% of OSCSCs x 100 **(A)**. Lytic units per 1 % NK cells were determined based on the percentages of CD16+/CD56+ NK cells in the cultures obtained by flow cytometric analysis **(B)**.
